# Exploring public perceptions and awareness of Parkinson’s disease: A scoping review

**DOI:** 10.1371/journal.pone.0291357

**Published:** 2023-09-15

**Authors:** Sophie Crooks, Gillian Carter, Christine Brown Wilson, Lisa Wynne, Patrick Stark, Michail Doumas, Matthew Rodger, Emma O’Shea, Gary Mitchell

**Affiliations:** 1 School of Nursing & Midwifery, Queen’s University Belfast, Belfast, Northern Ireland, United Kingdom; 2 Parkinson’s Association of Ireland, Dublin, Ireland; 3 School of Psychology, Queen’s University Belfast, Belfast, Northern Ireland, United Kingdom; 4 Centre for Gerontology and Rehabilitation, School of Medicine, University College Cork, Cork, Ireland; Swiss Paraplegic Research, SWITZERLAND

## Abstract

**Background:**

Parkinson’s disease (PD) is a common neurological disease affecting around 1% of people above sixty years old. It is characterised by both motor and non-motor symptoms including tremor, slow movement, unsteady gait, constipation and urinary incontinence. As the disease progresses, individuals living with the disease are likely to lose their independence and autonomy, subsequently affecting their quality of life. People with PD should be supported to live well within their communities but there has been limited research regarding what the public know about PD. This review aims to develop an understanding of how the public view people living with PD, which has the potential to aid the development of an educational resource for the future to improve public awareness and understanding of PD. The purpose of this scoping review is to review and synthesise the literature on the public perception and attitudes towards people living with PD and identify and describe key findings.

**Aim:**

This scoping review aims to explore public perceptions and awareness of Parkinson’s Disease among diverse populations, encompassing beliefs, knowledge, attitudes, and the broader societal context influencing these perceptions.

**Methods:**

A scoping review of the literature was conducted following the Preferred Reporting Items for Systematic Reviews and Meta-analysis extension for ScR (PRISMA-ScR). Four electronic databases were searched systematically (CINAHL Plus, Medline, PsycINFO and International Bibliography of the Social Sciences). The Joanna Briggs Institute Critical Appraisal Tools (JBI) were used to assess the quality of primary studies, however, all relevant studies were considered regardless of their methodological quality. The ‘Population-Concept-Context’ framework was used in the screening process to identify eligible papers.

**Results:**

A total of 23 studies were included in the review representing global research in quantitative (n = 12) and mixed methods approaches (n = 11). All 23 studies adopted some aspect of cross-sectional design. Three themes emerged from the studies, the first being public knowledge of symptoms, causes and treatment of PD and this highlighted a lack of understanding about the disease. Secondly, the review identified public attitudes towards PD, highlighting the social consequences of the disease, including the association between PD and depression, isolation and loss of independence. Finally, the third theme highlighted that there was a paucity of educational resources available to help increase public understanding of PD.

**Conclusion:**

Findings from this scoping review have indicated that public awareness of PD is a growing area of interest. To our knowledge, this is the first scoping review on this topic and review findings have indicated that public knowledge and attitudes towards PD vary internationally. The implications of this are that people with PD are more likely to be a marginalised group within their communities. Future research should focus on understanding the perception of the public from the perspective of people with PD, the development of interventions and awareness campaigns to promote public knowledge and attitude and further high-quality research to gauge public perceptions of PD.

## Introduction

Parkinson’s Disease (PD) is the fastest growing neurological condition in the world and is the second most common neurological condition after dementia [1, 2]. It is estimated that around 145,000 people in the United Kingdom (UK) are living with PD, affecting approximately 1% of people aged sixty years or older [3]. Causes of PD have been widely debated, but genetics, environmental and lifestyle factors are thought to play a key role [4]. There are, however, several risk factors that increase the likelihood of developing the disease such as pesticide exposure, rural living and traumatic brain injury [5, 6]. Additionally, age is the single most influential risk factor, with most people with Parkinson’s being aged 50 and above [4]. Furthermore, the likelihood of being diagnosed with PD increases by 1.4 times in men compared to women, however the reason for this is unknown [3].

PD was first described as a ‘shaking palsy’ by Dr. James Parkinson in 1817 [7]. It is a progressive neurological condition caused by death of cells in a particular area of the brain called the substantia nigra, resulting in reduction of dopamine production [8, 9]. Dopamine is a neurotransmitter involved in movement, memory and other functions and acts as a messenger that transports signals between the brain and nervous system which control and co-ordinate body movements [10, 11]. This reduction in dopamine causes both motor and non-motor symptoms including tremor, unsteady gait, rigidity, slurred speech and hallucinations [12, 13]. As symptoms heighten, individuals living with PD progressively lose their independence and autonomy [14], leading to feelings of depression or social isolation [15–17]. This subsequently impacts their quality of life and places a greater responsibility on caregivers and family members [18].

Good medical care, including the use of medication, can help manage PD. Medications, such as Levodopa, are available for the management of PD symptoms. Timely access to medication is essential in PD to prevent exacerbation of symptoms [19, 20]. Other strategies such as physiotherapy and occupational therapy are available to help people with PD manage and live with the disease [21]. Adopting a holistic approach to managing PD is essential, not only taking physical needs into consideration but also the person’s emotional, social and spiritual needs. This may include social interventions in the context of support groups, dancing, and regular exercise programmes which have been shown to reduce depression and anxiety levels, improve physical symptoms and improve overall quality of life for people with PD [22–33]. This is important because people living with PD may also experience symptoms which make them less likely to want to or have the ability, to engage in community activities, putting them at risk of social isolation [34]. These social activities are key components of healthy ageing, enabling older people to live well in the community, and are important considerations for future research and care delivery [35].

While there have been positive outcomes associated with social interventions for people living with PD, implementation of these is inconsistent. One reason why this inconsistency exists is because the public may not be aware of the unique needs and capabilities of people living with PD. Public awareness and attitudes to disease are important as they have a direct influence on how people live within their communities. Attitudes and perceptions of disease can be both positive and negative but have the potential to affect the quality of life (QoL), as well as social, physical and psychosocial wellbeing for those living with the condition. People living with PD can experience negative perceptions or attitudes from the public due to the lack of awareness and misunderstanding of disease symptoms, which may exacerbate feelings of depression and social isolation and impacting QoL [34].

There appears to be a lack of empirical investigation on the public understanding and awareness of PD, alongside an absence of reviews that synthesise the existing literature on this topic. With the increasing prevalence of PD and younger onset of diagnosis, it is important that the public are educated about the disease and how it affects those living with it. Without good awareness and knowledge of PD, the public are less likely to be able to effectively support people with PD in their local communities and plan or deliver meaningful social interventions. The aim of this review is therefore to synthesize international evidence to determine the current public perceptions and awareness of PD. This scoping review also aims to identify gaps in the research and inform the development for future research or educational resources for PD awareness.

## Methods

This scoping review (ScR) used a systematic approach, following guidelines from Joanna Briggs Institute (JBI) [36], Arksey and O’Malley [37] and the Preferred Reporting Items for Systematic Reviews and Meta-analysis extention for ScR (PRISMA-ScR) [38]. This process involved establishing a research question, identifying relevant studies through development of eligibility criteria and selecting relevant studies. Data were then analysed and extracted with the results summarised and reported.

### Search strategy

The search strategy was developed with the review team including input from a subject librarian. To attain a greater understanding of the topic and scope existing literature, an initial search was completed via Google Scholar focusing on public awareness of PD. Four electronic databases were used to conduct the review, CINAHL Plus (EBSCOhost), Medline All (Ovid), PsycINFO (Ovid) and International Bibliography of the Social Sciences (ProQuest). These specific databases were chosen as their literature primarily focusses on nursing, medicine, psychology and social sciences. Databases were searched between 10-20^th^ June 2022. A combination of search terms was used depending on MeSH terms and database selection including key search terms related to “Parkinson’s disease” or “public” or “perception” (see S1 File for search terms). The final part of the search strategy was to review the reference lists of included research studies to determine if any other suitable studies could be identified as recommended by Horsley et al [39]. All studies identified were imported to Covidence software for screening (https://www.covidence.org/).

### Inclusion/Exclusion criteria

The Population, Exposure and Outcome (PEO) framework was used to develop inclusion criteria as recommended by Bettany-Salttikov and McSherry [40]. This review included all types of empirical studies and evidence reviews (e.g., systematic reviews, quantitative research, qualitative research and mixed methods research). While this scoping review only includes studies written in English, no geographical restrictions were applied. There were also no year restrictions applied. Members of the public who did not provide professional or informal care (e.g., as a carer) to a person living with Parkinson’s Disease were included. In cases where a sample contained both professionals and the public, data were extracted only from the public. Similarly, in cases where public perception or awareness about PD was examined alongside another condition, data were extracted only about PD. Please see Table 1 for a detailed breakdown of the inclusion and exclusion criteria of this scoping review.

**Table 1:** Inclusion and exclusion criteria.

Inclusion	Exclusion
Empirical research on public awareness, understanding and perception of PD. Literature review, thesis, dissertation or conference abstracts Relevant grey literature and charity organisation literature that presented empirical data. English language articles No time restrictions	Non-English language articles People apart from the public, for example, health professionals, such as doctors, nurses, healthcare assistants People with PD or people who care or had cared for others with PD Studies regarding other neurological conditions that do not include PD Studies focussing on professional awareness or public interventions or people with PD’s opinions of the public

### Study selection

The database results were exported to Covidence, and all abstracts were screened by two people independently (SC & GM). This step was repeated for full text screening of applicable papers. Conflicts throughout the screening process were resolved following discussion with another member of the team (PS).

### Data extraction

Data were extracted from the included papers in Covidence using JBI Template Source of Evidence Details [41]. Data extracted included details about participants, concepts, context, study methods and key findings relevant to the review question. This review includes papers mostly quantitative research, for example, cross-sectional or non-experimental studies. Therefore, it was deemed most appropriate to use JBI Critical Appraisal Tools [41] due to the well-structured design and availability of tools for various study types. Quality appraisal is not obligatory for scoping reviews [38]; nonetheless, all papers were incorporated in this review irrespective of their methodological quality. This approach was adopted to provide readers with an understanding of the evidence’s quality in the review.

### Data analysis

The chosen approach of data analysis for this review was narrative synthesis due to the ability to organise findings from all included studies. Firstly, studies were investigated for similarities and differences, relationships explored, and the strength of evidence and results were assessed. To enhance the ease of presenting a narrative synthesis, specific elements were grouped together into themes, allowing for a more organized and understandable presentation. Findings from the studies were then summarised in themes and explained using text and words [42, 43]. As recommended by Levac et al. [44] themes are discussed in the context of the intended outcome of the study, i.e., summarising and presenting the existing literature surrounding public awareness and perceptions about PD and highlight gaps in the literature.

## Results

**Fig 1:**
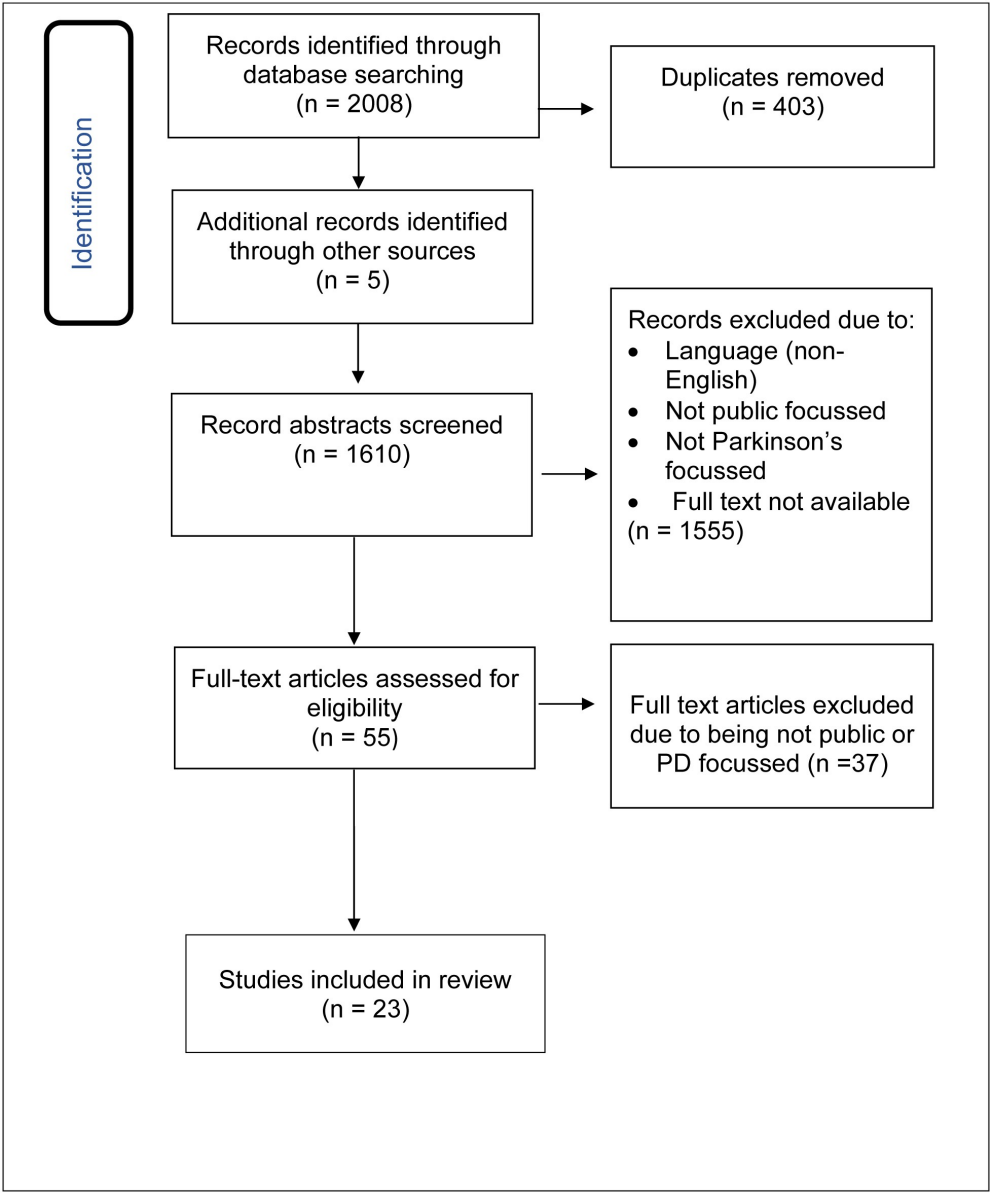
PRISMA-ScR flow chart. The databases of CINAHL Plus, Medline, PsycINFO, and Web of Science were searched and inclusion and exclusion criteria were included in the chart.

After the database search, a total of 2,008 papers were found. Fig 1 shows a PRISMA-ScR flow chart summarising the search process. Following abstract and title review, 55 papers remained for full text review. Three conflicts were resolved by consensus. In total 37 articles were excluded. Following this, five additional articles were included after hand searching reference lists. In total, 23 articles were included in the final review [45–67]. These studies were identified as eligible based on the inclusion criteria and on the primary aim for the review (Fig 1).

### Study characteristics

Twelve studies in this review used a quantitative approach, with the remaining eleven papers adopting a mixed methods approach. Details of study setting can be seen in Table 2. All 23 studies used some aspect of cross-sectional design. Eight studies were conducted in locations in Asia including Thailand [46], Malaysia [47], Saudi Arabia [48, 51], China [55], Israel [56] and South Korea [67]. Five took place in Canada [58–61, 63], three in the United States [49, 54, 64] and one in New Zealand [66]. The remaining four studies took place in Uganda [50], South Africa [53], Ireland [62], United Kingdom [65] and Australia [52]. One study by Chow and Viehweger [45] is a meta-analysis of eight primary research studies [57–64] completed in various locations worldwide. This meta-analysis by Chow and Viehweger [45] specifically concentrates on the youth population, whereas this review encompasses a broader range of demographics. Study settings varied from senior centres, tertiary care centres, health fairs, universities, online twitter accounts and within private homes.

**Table 2:** Study table.

Study	Sample size	JBI Score	Aim	Methods	Analysis	Key Findings
Alyamani *et al*. (2018) [48]–Asia	2609	8	To identify the level of awareness of our population regarding PD in terms of causes, signs, symptoms, and treatment.	Cross-sectional study using a questionnaire.	Descriptive statistics	Public knowledge of PD was moderate, recognising motor symptoms more than non-motor symptoms. Educational campaigns may be appropriate to improve public awareness of PD
Anpalagan *et al*. (2019) [57]—Asia	14	4	To determine the awareness of PD in Asia	Cross-sectional design using a questionnaire.	Descriptive statistics, fishers exact test	Young adults are adequately aware of the PD There is a need to increase the PD awareness among the young adult population through educational programs.
Chow and Viehweger (2019) [45]—Worldwide	167	4	To summarize the studies documenting the awareness of Parkinson’s disease.	Meta-analysis of 8 studies using questionnaires.	Descriptive statistics, fishers exact test	Young adults awareness of PD is adequate Educational programs could help achieve increased PD awareness and knowledge
Chow *et al*. (2019a) [58]—Canada	Not mentioned	4	To determine the awareness of PD in London, Canada.	Cross-sectional study using questionnaires.	Descriptive statistics, fishers exact test	Young adults are adequately aware of the disease Increased awareness among the young adult population could be achieved through educational programs.
Chow *et al* (2019b) [59]—Canada	41	4	To determine the awareness of PD in Canada.	Cross-sectional study using questionnaires.	Descriptive statistics, fishers exact test	Young adults awareness of PD is adequate Educational programs for young adults would be beneficial
Flynn *et al*. (2009) [66]–United Kingdom	126	4	To explore current public knowledge of aphasia and a comparator neurological condition (Parkinson’s disease). It also investigated respondents’ sources of information and whether demographic factors affected knowledge.	Cross-sectional study using questionnaires.	Not mentioned	Knowledge about PD was adequate, but better than knowledge of aphasia Respondents gained their knowledge mainly through the media and personal connections. There was evidence that ethnicity may affect knowledge.
Ho *et al*. (2019a) [60]—Canada	15	4	To determine the awareness of PD in Toronto, Canada.	Cross-sectional study using questionnaires.	Descriptive statistics, fishers exact test	Young adults are adequately aware of the disease Awareness among the young adult population could be increased through educational programs.
Ho *et al*. (2019b) [61]—Canada	26	4	To determine the awareness of PD in Halifax, Canada.	Cross-sectional study using questionnaires.	Descriptive statistics, fishers exact test.	Young adults are adequately aware of the disease Educational programs would increase awareness of PD in young adults
Hollenberg *et al*. (2019) [62]–Ireland	15	4	To determine the awareness of PD in Dublin, Ireland.	Cross-sectional study using questionnaires.	Descriptive statistics, fishers exact test	Young adults awareness of PD is adequate Increased awareness among the young adult population could be achieved through educational programs.
Jitkritsadakul *et al*. (2016) [46]—Asia	108	7	To evaluate general understanding of PD and identify knowledge gaps amongst PD patients using a validated PD knowledge questionnaire.	Cross-sectional study using questionnaires.	Chi-square, independent t-test, Durbin Watson test, Mann Whitney U test, Pearson’s correlation, logistic regression	Significant knowledge gaps were identified amongst PD patients in all three aspects of the questionnaire. Timely identification of patients with inaccurate or insufficient disease-related knowledge could help healthcare professionals choose more suitable multimodal educational interventions.
Kaddumukasa *et al*. (2015) [50]—Africa	377	7	To assess knowledge and attitudes towards PD in a Ugandan community sample.	Cross-sectional study using questionnaires.	Descriptive statistics, chi-square	PD is readily recognised in Uganda however, knowledge on causes is limited and misconceptions are common. Need for educational resources to improve understanding of PD and reduce stigma.
Khalifa *et al*. (2018) [51]—Asia	150	7	To determine the level of awareness among university students regarding Parkinson’s disease.	Cross-sectional study using questionnaires.	Chi-square	Most of the participants showed moderate to good knowledge about PD and positive attitudes toward PD, although there were deficits in some of the answers. Future research should continue to explore knowledge and attitudes about PD in diverse communities and different samples.
Landua. (2021) [49]–United States	206	4	To identify the current level of knowledge and attitudes about PD among college students enrolled at Oklahoma State University.	Cross-sectional study using questionnaires.	Not applicable	There is a significant need for more educational programs to be implemented to alleviate lack of knowledge, misconceptions and negative attitudes surrounding PD and its possible management.
McCann *et al*. (2013) [65]–New Zealand	300	4	To investigate whether public awareness of aphasia has improved since the original international study by Simmons-Mackie et al. in 2002. A second aim was to compare health professionals’ awareness and knowledge of aphasia specifically with that of the public.	Cross-sectional study using questionnaires.	Not mentioned	Ten years after the first surveys, awareness and knowledge of PD, stroke and aphasia remain low. Efforts need to be taken to increase awareness and knowledge of these conditions to increase the health outcomes for people with aphasia and their families.
Mokaya *et al*. (2017) [53]–South Africa	Not mentioned	4	To explore how PD is perceived and conceptualised within communities in a Xhosa speaking black population in South Africa.	Cross-sectional study using questionnaires.	Not mentioned	There is a lack of knowledge about PD among black South Africans Almost half of the respondents felt people with PD should not live within the community and a third considered witchcraft as a cause of PD Public education would make it easier for people with PD to adapt to their condition within the community
Moore and Knowles. (2006) [52]–Australia	200	6	To ascertain beliefs and attitudes about PD, specifically the extent of negative attitudes, stereotyping and stigma associated with the disease.	Cross-sectional study using two questionnaires.	Descriptive statistics	Overall knowledge of PD was good, however, almost half of the respondents saw a stigma attached to PD. Highlights the need for public education, alongside programs for people with early onset PD.
Pan *et al*. (2014) [54]–United States	229	8	Understand knowledge and attitudes about PD among a racially/ethnically diverse group of community members.	Cross-sectional study involving focus groups (qualitative) and administration of a questionnaire (quantitative).	Logistic regression, Comparative analysis method	There is a low level of knowledge of PD in older adults Main barriers to care include fear, stigma and communication barriers Need for educational campaigns for PD was highlighted
Tan *et al*. (2015) [47]—Asia	1285	8	To explore the level of public knowledge regarding PD in a large multi-ethnic urban Asian cohort, and (as a secondary aim) in a smaller cohort of PD patients and caregivers.	Cross-sectional study using questionnaires.	Chi-square, analysis of variance, preliminary assumption testing	Important gaps in knowledge of PD were evident The need for further research and specific educational campaigns in this area was highlighted
Viehweger *et al*. (2019a) [63]—Canada	19	4	To determine the awareness of PD in Waterloo, Canada.	Cross-sectional study using questionnaires.	Descriptive statistics, fishers exact test	Young adults are adequately aware of the disease Educational programs could achieve increased awareness among this population
Viehweger *et al*. (2019b) [64]–United States	13	4	To determine the awareness of PD in New York, USA	Cross-sectional study using questionnaires.	Descriptive statistics, fishers exact test	Young adults knowledge of PD is sufficient Awareness of PD could be achieved through educational programs.
Werner and Korczyn. (2010) [56]—Asia	632	4	To assess the prevalence and socio-demographic correlates of lay persons’ beliefs and knowledge about PD.	Cross-sectional study using questionnaires.	Descriptive statistics, chi-square, spearman’s correlation, logistic regression, Mann Whitney U test	Low number of participants reported knowing much about PD and reported being worried or fearful of getting PD. Highlighted the need for expansion of PD research, alongside development of programs aiming to disseminate knowledge about PD.
Youn *et al*. (2016) [67]—Asia	1000	7	To investigate the awareness and knowledge about PD among the general population in South Korea and to identify the factors that are associated with these parameters	Cross-sectional study using questionnaires.	Logistic regression	Knowledge of younger subjects exhibited the least knowledge compared to respondents over 40 years of age. Low socioeconomic status was associated with poor knowledge of PD. Awareness and knowledge of PD showed hierarchical gradients with respect to age, income and education level. Educational strategies and approaches targeting specific subgroups are necessary to improve public awareness and knowledge about PD.
Zhang *et al*. (2018) [55]—Asia	238	6	To determine the rate of awareness, drug treatment and rehabilitation in PD.	Analysis and summary of data from Prevention and Intervention on Neurodegenerative Disease for the Elderly in China (PINDEC).	Descriptive statistics, chi-square	Rates of awareness, drug treatment and rehabilitation for elderly PD patients were low in China. Efforts to increase health education for PD should be made among the elderly.

### Sample

Samples sizes range from 13 to 2609 participants, with a combined total of 3,701 participants in this review. Demographic information of participants can be seen in S2 File.

### Data collection

Questionnaires were used by 19 studies, using face-to-face, online or telephone methods [46–49, 51–54, 57–67]. Seven studies used questionnaires that had been previously tested, validated and replicated [47, 48, 51, 52, 54, 65, 67]. The remaining studies were unclear about how the questionnaire was developed. Questionnaires were clearly shown within tables or appendices within 16 studies [46–49, 51, 52, 54, 57–65]. One study [54] adopted a mixed methods approach using both questionnaires and focus groups. A face-to-face interview method was conducted by one study [54] and two studies conducted telephone interviews [50, 56]. Questionnaires were used collect quantitative data via open and closed questions in all papers, apart from one [54] which used a qualitative questionnaire for data collection. Data was analysed using a mixture of qualitative and quantitative approaches.

### Quality appraisal

The quality of papers was appraised using the JBI source of evidence details critical appraisal tool. Quality appraisal is not mandatory for scoping reviews [38]; nevertheless, all papers were included regardless of their methodological quality to inform readers about the evidence’s quality. Details of quality appraisal for each paper are shown in Study Table 2. Each paper used some aspect of cross-sectional design, therefore the JBI Analytical Cross Sectional Studies Checklist was used [68]. A scoring system out of 8 was used, with total scores of 0–4 deemed low quality, 5–6 deemed average quality and 7–8 high quality. Scores were given based on the number of ‘yes’ responses. Overall, seven papers were deemed high quality [46–51, 54, 67], three were average quality [52, 55, 57] and the remaining 13 papers were deemed low quality [45, 49, 53, 56, 58–66]. Papers that were deemed low quality had the underlying issue of low sample size and not using a previously validated questionnaire for data collection.

### Study results

This review has explored the public perceptions and awareness of Parkinson’s Disease. In total three themes emerged following narrative synthesis. To facilitate a narrative synthesis, findings were grouped into themes, resulting in a more organized and coherent presentation [69]. This process involved data familiarisation, data coding and identification of patterns or similarities. This allowed themes to be generated that represented overarching ideas or concepts.

The first theme addresses the level of knowledge the public had about the main symptoms of PD including tremor and difficulties with communication, alongside causes, risk factors and available treatments for the disease. The second theme focusses on public attitudes towards PD, discussing its social consequences and its psychosocial impact including the association with depression, isolation and loss of independence. Lastly, the importance of raising awareness of PD is the third theme identified.

### Theme one—Public knowledge of symptoms, causes and treatment of Parkinson’s disease

Evidence from the papers in this review shows public knowledge of symptoms, causes and treatment of PD is adequate, however, most studies highlighted the benefits further research and education in this area would have for knowledge and awareness of PD. The level of knowledge of symptoms of PD was a key topic that was highlighted throughout the literature where participants commonly attributed symptoms of PD to ageing, as opposed to disease. Several studies highlighted that motor symptoms were significantly more known that non-motor symptoms [46–49]. In particular, the most identified motor symptom was tremor. One study [49] also reported a large amount (n = 154) (75%) of participants who identified communication issues as a symptom of PD, and around two thirds of participants were aware of mobility problems.

Findings from this review also indicate that the causes of PD are also poorly understood. One study [50] reported that participants were unaware of what part of the body PD affects, however, three studies [47, 48, 51] discuss how participants were aware that it was a neurological condition affecting the brain and dopamine production. In contrast, another study by Moore and Knowles [52] reported more than half of respondents appreciated that the exact cause is unknown. Despite this, studies included in the review failed to determine public knowledge of aetiology or risk factors of PD however, one study [53] highlighted that witchcraft was a perceived cause of PD according to some public perceptions in Africa.

A lack of awareness of treatment for PD was an aspect that came up frequently in the literature. Two studies [46, 54] revealed a lack of awareness of medication for PD, with almost half of the respondents in one study holding the belief there is a cure [46]. Additionally, one study revealed a lack of awareness of alternative treatments such as deep brain stimulation and stem cell transplants for the treatment of PD [46]. On the other hand, most participants in four studies [47, 48, 52, 55] were aware there are treatments available for PD that improve symptoms and that treatment is lifelong. These findings were positive as most of the public in the reported studies had some knowledge of what PD is, that medications are available to help symptoms and that continual treatment is required.

Overall, this theme illustrates that the public have variable knowledge about Parkinson’s Disease. While many were aware of the condition, there was poor awareness about the non-motor symptoms and advanced/device-assisted medical treatments for PD.

### Theme two—Public attitudes towards Parkinson’s disease

Attitudes towards disease, including PD, are essential as they contribute to determining the environment which the person lives and interacts. Poor attitudes towards PD could be a result of the lack of knowledge of the disease as identified in theme one. Findings relating to public attitudes included social consequences and perceptions about quality of life for people with PD with associations to depression, isolation and loss of independence.

In this review, five studies identified areas of social consequences surrounding PD in the public. These took the form of being worried or fearful of developing PD [56], not wanting to have a relationship with someone with PD [49, 51], being a burden to others [54, 56] and the belief that PD is contagious or is a form of insanity [50]. For example, in one study [49] around 30% (n = 62) of participants reported they would not marry someone with PD.

Several studies reported that just over half of participants linked depression to PD [45, 50, 54, 57–64] For example, over 50% (n = 190) of participants in one study [50] held the belief that depression is common in those with PD. Similarly, in a meta-analysis of eight studies [58], 51% (n = 20) of participants believed there to be a correlation between PD and depression.

Participants also claimed they were aware that people with PD often feel socially isolated [47, 48]. The link between PD and isolation was also discussed in ten other studies in the review [45, 54, 57–64]. In one study, 78% (n = 1002) of participants agreed that people with PD often feel socially isolated [47]. This is reaffirmed by Alyamani et al [48], where 75% (n = 1956) of participants made links between PD and social isolation. In addition to this, participants in two studies [49, 54] highlighted the loss of independence people with PD face and 62% (n = 142) of participants in Pan et al [54] study have the perception that a PD diagnosis would be difficult to accept. Khalifa et al [51] state more than half (n = 78) of the study participants felt supportive towards people with PD and participants within Landua’s [49] study had an overall compassionate and sympathetic view towards those with PD.

Overall, this theme has highlighted public attitudes towards PD, particularly the psychosocial impact of living with the disease. Some studies have also demonstrated positive public attitudes towards those with PD, expressing feelings of supportiveness, compassion and sympathy.

### Theme three—Raising awareness of Parkinson’s disease

Findings of twenty-one studies included in this review acknowledged the benefits of raising awareness of PD [45–50, 52–67]. For example, three studies highlight the advantages of raising awareness through campaigns and advocacy programmes, could have the beneficial effect of reducing health disparities by facilitating earlier diagnosis and treatment, building community supports and reducing stigma [47, 50, 54] with the overall aim of empowering and supporting those with PD to live happily with a great quality of life.

Most studies within the review discussed the importance of implementing resources for the public to be educated on PD [45–50, 53–55, 58–65]. Although no study specifically gave suggestions of how this could be done, they all highlighted the benefits of increased public awareness of the disease, with the aim to create supportive and encouraging communities for people with PD.

## Discussion

This scoping review aimed to summarize and present the current literature on public awareness and perceptions of PD, while also identifying gaps in the existing research. This review identified the level of public knowledge and significant misconceptions about PD including its symptoms, aetiology, and prognosis. This is more pertinent in developing and less developed countries [50, 53] compared to developed countries [48, 51, 57–61, 63]. Most of the PD awareness revolves around motor symptoms as opposed to non-motor symptoms [48, 49, 70, 71].

According to literature, the problem of poor awareness and lack of knowledge in general is associated with several factors. Poor health literacy may be a key factor impacting public knowledge of not only PD, but other health related issues. Health literacy refers to the ability individuals have to obtain, process and understand basic health information to make appropriate health decisions [72]. Poor health literacy may also have links to the country where the studies took place. Research has suggested links between poor health literacy and many low-and middle- income countries due to low levels of general literacy, alongside poorly resourced and functioning health systems [73]. Two studies [50, 53] in this review took place in low-middle income countries (Uganda and South Africa) and have demonstrated a lack of knowledge and greater misconceptions surrounding PD, compared with studies in high-income countries including Saudi Arabia [48, 51], Taiwan and Singapore [57] and Canada [58–61, 63], who demonstrated adequate knowledge about PD. Also, one study [67] associated low socioeconomic status with a lack of knowledge of PD. Additionally, previous research has shown older age to be significantly associated with low health literacy [74, 75]. Older people are a high priority group in society for improving health literacy as they are likely to be motivated to increase their knowledge so they can recognise PD in spouses or friends of a similar age. The age of participants in this review ranges from 18–88 years old, with the average age being 53 years old. Low health literacy can also be associated with limitations in ability to access information within these groups, for example, less health education, and limited access to internet and information facilities [76, 77].

Data about attitudes towards PD were present in the reviewed literature in the form of analysis of social consequences and perceptions about quality of life in PD. Firstly, the link between PD and depression was apparent in the literature which has the potential to influence public attitudes towards people with PD. In addition to this, many studies within the review made links to PD and social isolation [45–48, 54, 57–64]. Similarly, Subramanian et al [34] who concluded that social isolation has close links to PD severity. It is, however, not clear if this is a cause or consequence of the disease, or even if the relationship is cyclical. Loss of independence, difficulty accepting diagnosis and feelings of being a burden to others were among other social consequences attached to PD in this review [49–51, 54, 56]. The public are aware that living with PD can cause social problems impacting quality of life, potentially leading to depression, loneliness and other negative psychosocial consequences. On the other hand, increasing public awareness of these social consequences of PD may encourage the public to be understanding and supportive to those living with PD, helping them to live well within their communities.

Social consideration and attitudes towards PD are essential since they contribute to determining the environment in which a person with PD lives and interacts [52]. Better understanding of the disease for the public moves a step towards tailored person-centredness, enabling others to see people with PD as a person living an everyday life. The Michael J Fox Foundation leads PD research in the US, seeking a cure and raising awareness to reduce stigma through fundraising and advocacy [78]. However, there is a lack of similar interventions and campaigns in the UK PD community. To initiate the process of improving public knowledge and awareness of PD and eradicate stigma, education is essential. Education was identified as critical to improve knowledge and raise awareness of PD in this review. Similarly, others suggest ways in which public education in PD can be improved. Examples include integrated media such as short films [79], electronic learning modules and webinars [80] and use of social media to disseminate knowledge [81] which have increased public understanding of PD and other conditions. Including and consulting people with PD in the design process of such campaigns and educational resources has proven effective for allowing others to see through the ‘lens’ of people with PD and resources become easier to implement by reducing the translation gap [82].

To shape active aging as a lifelong process and recognise the potential that older people represent in society, the World Health Organisation challenged communities worldwide to become more age friendly [83]. Building age-friendly communities has been proven to improve life satisfaction of the older person, provide feelings of security, and sense of autonomy and independence [84]. A review by Tavares et al. [85] found growth of age-friendly community initiatives to be positively associated with social participation, community support and greater life satisfaction. Given the level of public knowledge and awareness demonstrated in this review, future research may focus on the development of a Parkinson’s specific framework. Development of such resources can help support those with the disease to live normal, healthy lives within their community given the success of age-friendly communities.

An interesting omission in the included studies worth noting was the link between PD and palliative care. Various studies over recent years have highlighted the lack of public knowledge and negative perceptions towards palliative care, particularly in those without family-related experiences [86–88]. Increased awareness of palliative care and advance care planning is needed amongst all members of the public to improve knowledge and access to services, empower individuals, involve communities and develop international strategies [87–89]. It was therefore surprising to note that none of the included papers explored any aspect of public awareness of palliative care or advance care planning and the association to PD. Given the recent focus on enhancing public awareness about palliative care and advanced care planning as well as the paucity of research on this found in the present review, it is recommended that future interventional research about PD education or public awareness should address this area.

### Strengths and limitations

A main strength is that, to our knowledge, this is the first time a scoping review aiming to explore public perceptions and awareness of PD has been conducted. Due to time and resource constraints, only four databases were chosen to complete the search. While this is common within scoping reviews, the use of a larger number of databases may have yielded a greater number of papers. However, the use of broad search terms with precise inclusion and exclusion criteria ensured we were able to capture as many papers as possible within these databases to answer our research question. A further limitation was we only included papers written in English and papers in other languages may have provided additional data.

## Conclusion

Findings from this review have indicated that public awareness of PD is a growing area of interest. Review findings suggest that public knowledge and attitudes vary across the globe. The implications of this are that people with PD are more likely to be a marginalised group within their communities, despite this being a common disease of older people. Future research should focus on understanding the perception of the public from the perspective of people with PD, the development of interventions to promote public knowledge and attitude and further high-quality research to gauge public perceptions of PD internationally.

## Supporting information

S1 File Combination of key search terms (PEO). (DOCX)

S2 File Characteristics of participants. (DOCX)

S1 Checklist PRISMA-P (Preferred Reporting Items for Systematic review and Meta-Analysis Protocols) 2015 checklist: Recommended items to address in a systematic review protocol*. (DOC)
