# Supplementary material for: Exploring public perceptions and awareness of Parkinson’s disease: A scoping review
Source: PLoS One. 2023 Sep 15;18(9):e0291357. doi: 10.1371/journal.pone.0291357 (PMC10503766; doi:10.1371/journal.pone.0291357)
Supplement: S1 File — (DOCX) [file pone.0291357.s001.docx]

**Supplementary File 1 – Combination of key search words (PEO)**

**Key Words (P)**

Parkinson

**OR**

Parkinson’s disease

**OR**

PD

**OR**

Progressive supranuclear palsy

**OR**

Multiple system atrophy

**OR**

Corticobasal degeneration

**Key Words (E)**

Dementia friendly*

**OR**

Age friendly*

**OR**

Senior Friendly*

**OR**

Communit*

**OR**

Community network

**OR**

Social participation

**OR**

Social inclusion

**OR**

Social health

**OR**

Social integration

**OR**

Public*

**OR**

Young people

**OR**

Young*

**OR**

Child*

**OR**

Children

**OR**

Adolescent

**Key Words (O)**

Understanding

**OR**

Awareness

**OR**

Perception

**OR**

Knowledge

**OR**

Experiences

**AND**

**AND**

| **Combination of key search words (PEO)** |
| --- |
