# Supplementary material for: Exploring public perceptions and awareness of Parkinson’s disease: A scoping review
Source: PLoS One. 2023 Sep 15;18(9):e0291357. doi: 10.1371/journal.pone.0291357 (PMC10503766; doi:10.1371/journal.pone.0291357)
Supplement: S2 File — (DOCX) [file pone.0291357.s002.docx]

**Supplementary File 2 – Characteristics of Participants**

**Characteristics of participants**

| **Study** | **Age (range)** | **Gender** | **Race** | **Education** | **Healthcare education/occupation** |
| --- | --- | --- | --- | --- | --- |
| Alyamani et al (2018) | 25.7 (average) | F – 59%  M – 41% | NA | Primary – 0.21%  Secondary – 21%  Tertiary – 74.5%  Higher – 4.5% | 32.5% |
| Chow & Viehweger (2019) | 18-22+ | F – 75%  M – 23%  Not mentioned – 2% | NA | NA | 21% |
| Flynn et al (2009) | 18-70+ | F -52%  M – 48% | White British – 73%  White other – 14%  African British – 2%  African other – 4%  Asian British – 3%  Asian other – 4% | NA | NA |
| Jitkritsadakul et al (2016) | 50-72 | F – 96%  M – 4% | NA | Primary + secondary – 34%  Tertiary – 51%  Higher – 15% | NA |
| Kaddumukasa et al (2015) | 18-85 | F – 67.4%  M – 32.6% | NA | NA | NA |
| Khalifa et al (2018) | 18-35 | F – 90%  M – 10% | NA | Tertiary – 100% | NA |
| Landua (2021) | 18-49 | F – 58.7%  M – 41.2% | White – 78%  Hispanic – 6%  Asian – 5% | NA | NA |
| McCann et al (2013) | 44 (average) | F – 58.3%  M – 41.6% | NA | NA | 30% |
| Mokaya et al (2017) | NA | NA | NA | NA | NA |
| Moore and Knowles (2006) | 18-50+ | F – 63%  M – 37% | NA | Primary – 22%  Secondary – 20%  Tertiary – 17%  Higher – 41% | NA |
| Pan et al (2014) | 71-76 | F – 70%  M – 30% | White - 37%  African American – 36%  Chinese American – 27% | NA | NA |
| Tan et al (2015) | 37 (average) | F – 53%  M – 47% | Chinese – 61%  Malaysian – 24%  Indian – 11%  Other – 4% | None – 0.5%  Primary – 1.2%  Secondary – 23.1%  Tertiary – 75.2% | 28.3% |
| Werner & Korczyn (2010) | 18-88 | F – 52.5%  M – 47.5% | NA | 14 years (average) | NA |
| Youn et al (2016) | 18-60+ | NA | NA | 12+ years | NA |
| Zhang et al (2018) | 60-80+ | F – 51.2%  M – 48.7% | NA | NA | NA |
