# Supplementary material for: Exploring public perceptions and awareness of Parkinson’s disease: A scoping review
Source: PLoS One. 2023 Sep 15;18(9):e0291357. doi: 10.1371/journal.pone.0291357 (PMC10503766; doi:10.1371/journal.pone.0291357)
Supplement: S1 Checklist — (DOC) [file pone.0291357.s003.doc]

**PRISMA-P (Preferred Reporting Items for Systematic review and Meta-Analysis Protocols) 2015 checklist: recommended items to address in a systematic review protocol***

| Section and topic | Item No | Checklist item |
| --- | --- | --- |
| ADMINISTRATIVE INFORMATION | | |
| Title: |  |  |
| Identification | 1a | A protocol of a scoping review |
| Update | 1b | N/A |
| Registration | 2 | N/A |
| Authors: |  |  |
| Contact | 3a | Sophie Crooks^1*^, Queen’s University Belfast, School of Nursing & Midwifery, Belfast, Northern Ireland, UK.  [Scrooks08@qub.ac.uk](mailto:Scrooks08@qub.ac.uk), Medical Biology Centre, 97 Lisburn Road, Belfast, Northern Ireland, BT7 BL (corresponding author)  Gillian Carter^1^, Queen’s University Belfast, School of Nursing & Midwifery, Belfast, Northern Ireland, UK.  [g.carter@qub.ac.uk](mailto:g.carter@qub.ac.uk)  Christine Brown Wilson^1^, Queen’s University Belfast, School of Nursing & Midwifery, Belfast, Northern Ireland, UK.  [c.brownwilson@qub.ac.uk](mailto:c.brownwilson@qub.ac.uk)  Lisa Wynne^2^, Parkinson’s Association of Ireland, Dublin, Ireland  [nurse@parkinsons.ie](mailto:nurse@parkinsons.ie)  Patrick Stark^1^, Queen’s University Belfast, School of Nursing & Midwifery, Belfast, Northern Ireland, UK.  [p.stark@qub.ac.uk](mailto:p.stark@qub.ac.uk)  Michail Doumas^3^, Queen’s University Belfast, School of Psychology, Belfast, Northern Ireland, UK.  [m.doumas@qub.ac.uk](mailto:m.doumas@qub.ac.uk)  Matthew Rodger^3^, Queen’s University Belfast, School of Psychology, Belfast, Northern Ireland, UK.  [m.rodger@qub.ac.uk](mailto:m.rodger@qub.ac.uk)  Emma O’Shea^4^, Centre for Gerontology and Rehabilitation, School of Medicine, University College Cork, Cork, Ireland.  [emma.oshea@ucc.ie](mailto:emma.oshea@ucc.ie)  Gary Mitchell^1^, Queen’s University Belfast, School of Nursing & Midwifery, Belfast, Northern Ireland, UK.  [gary.mitchell@qub.ac.uk](mailto:gary.mitchell@qub.ac.uk) |
| Contributions | 3b | All authors have agreed the final version of this paper and agree to be accountable for this review. All authors have also met criteria adapted from McNutt et al. Proceedings of the National Academy of Sciences, Feb 2018, 201715374. <https://doi.org/10.1073/pnas.1715374115>; licensed under CC BY 4.0. All authors been involved in substantial contributions to the conception (SC, GC, CBW, PS, LW, EOS, MR, MD, GM), design of the work (SC, GC, CBW, PS, LW, EOS, MR, MD, GM), the acquisition, analysis, interpretation of data (SC, GM), have drafted the work or substantively revised it (SC, GC, CBW, PS, LW, EOS, MR, MD, GM). The authors read and approved the final manuscript (SC, GC, CBW, PS, LW, EOS, MR, MD, GM). |
| Amendments | 4 | N/A |
| Support: |  |  |
| Sources | 5a | This review has received no financial support |
| Sponsor | 5b | This review has received no funding or sponsors |
| Role of sponsor or funder | 5c | N/A |
| INTRODUCTION | | |
| Rationale | 6 | There appears to be a lack of empirical investigation on the public understanding and awareness of PD. With the increasing prevalence of PD and younger onset of diagnosis, it is important that the public are educated about the disease and how it affects those living with it. Without good awareness and knowledge of PD, the public are less likely to be able to effectively support people with PD in their local communities and plan or deliver meaningful social interventions. The aim of this review is therefore to synthesize international evidence to determine the current public perceptions and awareness of PD. |
| Objectives | 7 | This review aims to explore public perceptions and awareness of Parkinson’s Disease (PD). |
| METHODS | | |
| Eligibility criteria | 8 | This review included all types of empirical studies and evidence reviews (e.g., systematic reviews, quantitative research, qualitative research and mixed methods research). Members of the public who did not provide professional or informal care (e.g., as a carer) to a person living with Parkinson’s Disease were included. In cases where a sample contained both professionals and the public, data were extracted only from the public. Similarly, in cases where public perception or awareness about PD was examined alongside another condition, data were extracted only about PD. |
| Information sources | 9 | An initial search was completed via Google Scholar focusing on public awareness of PD. Four electronic databases were used to conduct the review, CINAHL Plus (EBSCOhost), Medline All (Ovid), PsycINFO (Ovid) and International Bibliography of the Social Sciences (ProQuest). Databases were searched between 10^th^ June 2022 – 20^th^ June 2023. |
| Search strategy | 10 | Search terms include Parkinson **OR** Parkinson’s disease **OR** PD **OR** Progressive supranuclear palsy **OR** Multiple system atrophy **OR** Corticobasal degeneration **AND** dementia friendly* **OR** age friendly* **OR** senior friendly* **OR** community* **OR** community network **OR** social participation **OR** social inclusion **OR** social health **OR** social integration **OR** public* **OR** young people **OR** young* **OR** child* **OR** children **OR** adolescent **AND** understanding **OR** awareness **OR** perception **OR** knowledge **OR** experiences.  Only studies written in English were include and no geographical restrictions were applied. There were also no year restrictions applied. |
| Study records: |  |  |
| Data management | 11a | The database results were exported to Covidence, and all abstracts were screened by two people independently (SC & GM). |
| Selection process | 11b | The database results were exported to Covidence, and all abstracts were screened by two people independently (SC & GM). This step was repeated for full text screening of applicable papers. Conflicts throughout the screening process were resolved following discussion with another member of the team (PS). |
| Data collection process | 11c | Data were extracted from the included papers in Covidence using JBI Template Source of Evidence Details. Data extracted included details about participants, concepts, context, study methods and key findings relevant to the review question. This review includes papers mostly quantitative research, for example, cross-sectional or non-experimental studies. Therefore, it was deemed most appropriate to use JBI Critical Appraisal Tools due to the well-structured design and availability of tools for various study types. While quality appraisal is not mandatory for scoping reviews, this step was undertaken to provide readers of this review with an understanding of evidence quality. |
| Data items | 12 |  |
| Outcomes and prioritization | 13 |  |
| Risk of bias in individual studies | 14 | N/A |
| Data synthesis | 15a | The chosen approach of data analysis for this review was narrative synthesis due to the ability to organise findings from all included studies. Firstly, studies were investigated for similarities and differences, relationships explored, and the strength of evidence and results were assessed. Findings from the studies were then summarised in themes and explained using text and words (42). Themes are discussed in the context of the intended outcome of the study, i.e., determining public awareness and perceptions about PD |
|  | 15b | N/A |
|  | 15c | N/A |
|  | 15d | N/A |
| Meta-bias(es) | 16 | N/A |
| Confidence in cumulative evidence | 17 | The quality of papers was appraised using the JBI source of evidence details critical appraisal tool. Details of quality appraisal for each paper are shown in Study **Table 2**. A scoring system out of 8 was used, with total scores of 0-4 deemed low quality, 5-6 deemed average quality and 7-8 high quality. |

*** It is strongly recommended that this checklist be read in conjunction with the PRISMA-P Explanation and Elaboration (cite when available) for important clarification on the items. Amendments to a review protocol should be tracked and dated. The copyright for PRISMA-P (including checklist) is held by the PRISMA-P Group and is distributed under a Creative Commons Attribution Licence 4.0.**

*From: Shamseer L, Moher D, Clarke M, Ghersi D, Liberati A, Petticrew M, Shekelle P, Stewart L, PRISMA-P Group. Preferred reporting items for systematic review and meta-analysis protocols (PRISMA-P) 2015: elaboration and explanation. BMJ. 2015 Jan 2;349(jan02 1):g7647.*
